# Total knee arthroplasty and femoral osteotomy with a patient-specific cutting guide to treat haemophilic arthritis with severe extra-articular deformity: A case report and review of literatures

**DOI:** 10.3389/fsurg.2022.1067306

**Published:** 2023-01-06

**Authors:** Shuai-Jie Lv, Zheng-Ming Wang, Rui Wang, Heng-Kai Jin, Pei-Jian Tong, Xun Liu

**Affiliations:** ^1^ Orthopedics and Traumatology, The First Affiliated Hospital of Zhejiang Chinese Medical University(Zhejiang Provincial Hospital of Chinese Medicine), Hangzhou, China; ^2^ Shi's Center of Orthopedics and Traumatology, Shuguang Hospital Affiliated to Shanghai University of Traditional Chinese Medicine, Shanghai, China; ^3^ The First School of Clinical Medicine, Zhejiang Chinese Medical University, Hangzhou, China

**Keywords:** haemophilic arthritis, extra-articular deformity, PSI, total knee arthroplasty, case report

## Abstract

**Background:**

Total knee arthroplasty (TKA) is recommended for haemophilic patients with end-stage arthritis. TKA combined with a one-stage extraarticular osteotomy is uncommon in the treatment of haemophilic arthritis (HA) with severe extra-articular deformities (EADs) and a history of inhibitors under the guidance of a patient-specific cutting guide (PSI).

**Case presentation:**

We reported a 20-year-old male patient with severe haemophilia, limited knee functionality, a 30° sagittal deformity on the femoral side and a history of inhibitors. We adopted the Van Creveld protocol to decrease the inhibitors. TKA and extra-articular osteotomy (EAO) were performed simultaneously and sequentially under the guidance of PSI. An appropriate central alignment of the lower limb was restored by using cement prostheses with antibiotics and femur shaft locking compression plates. The last follow-up showed that the knee function was good, the VAS score was 0, the WOMAC score was 18 and the ROM was 0°–95°.

**Conclision:**

Regular haematology management can reduce the perioperative bleeding risk in haemophilic patients treated with inhibitors. PSI plays an important role in guiding the TKA and EAO of end-stage HA patients with severe EAD.

## Introduction

More than 80% of bleeding in haemophilia patients occurs in the musculoskeletal system (1). Repeated spontaneous bleeding leads to irreversible damage to the joints, which eventually progresses to haemophilic arthropathy (HA). As HA progresses, pain, loss of joint movement, and progressive deformities will become apparent, which seriously affect the quality of life of haemophilia patients. Due to their coagulation dysfunction and the high cost of coagulation factors, some patients receive nonsurgical treatment after fractures, which may lead to the occurrence of an extra-articular deformity (EAD).

EAD can change the distribution of the mechanical load on the knee joint and accelerate the occurrence of arthritis (2). Total knee arthroplasty (TKA) is challenging due to the influences of the abnormal stress, anatomical structures, and soft tissue conditions for HA with severe EAD. Surgical options include a TKA combined with extra-articular osteotomy (EAO) or an intra-articular compensatory osteotomy (3). Most scholars (4–6) believe that a TKA combined with an EAO should be considered when the angle of coronal or sagittal deformity on the femoral side is greater than 20 to avoid an excessive intra-articular osteotomy, a poor mechanical axis (MA) and joint instability. Patient-specific cutting guides (PSIs) provide a new solution for HA with severe EADs during TKA (6).

We report a case of a patient with severe haemophilia, sagittal deformity (30°) on the femoral side and a history of inhibitor usage.

## Case presentation

A 20-year-old male presented with pain and limited motion in the right knee for 15 years. The patient presented to our clinic with HA, restricted flexion and extension of the right knee and a sagittal deformity of the right femur 8 months prior to the surgery. He was diagnosed with severe haemophilia A (coagulation factor VIII was 1.52%) and haemophilic arthritis of right knee. At the age of 13, the patient was fixed with plaster for a fracture of the femoral shaft, which resulted in a malunion.

Because he was positive for inhibitors, the patient was re-examined regularly by the Haematology Clinic after discharge. The patient did not take any interventions during the first 4 months while being positive for inhibitors. During this period, the patient suffered from knee joint bleeding and recovered after ice compression and lying in bed. In the 5th month after the discovery of the inhibitors, the patient received irregular injections of factor VIII intravenously (1,000 IU, BIW or TIW, Kovaltry, Bayer), which did not follow the haematologist's recommendation. After 66 days of injections, he was still positive for inhibitors. Since then, the patient started to receive regular injections of factor VIII (1,500 IU, BID) intravenously. Forty-nine days after the first injection, he became negative for inhibitors (Table 1). The patient came to the hospital again 3 days prior to the surgery.

**Table 1:** Monitoring value of FVIII inhibitor and interventions.

Date	FVIII inhibitor/BU	Interventions
2019.03.18	3.7%	None
2019.05.09	3.5%	None
2019.07.04	2.8%	None
2019.07.25	2.1%	1,000 IU, iv, BIW-TIW
2019.08.08	2.1%	1,000 IU, iv, BIW-TIW
2019.09.12	2.2%	1,500 IU, iv, BID
2019.10.31	0.6%	

**Figure 1:**
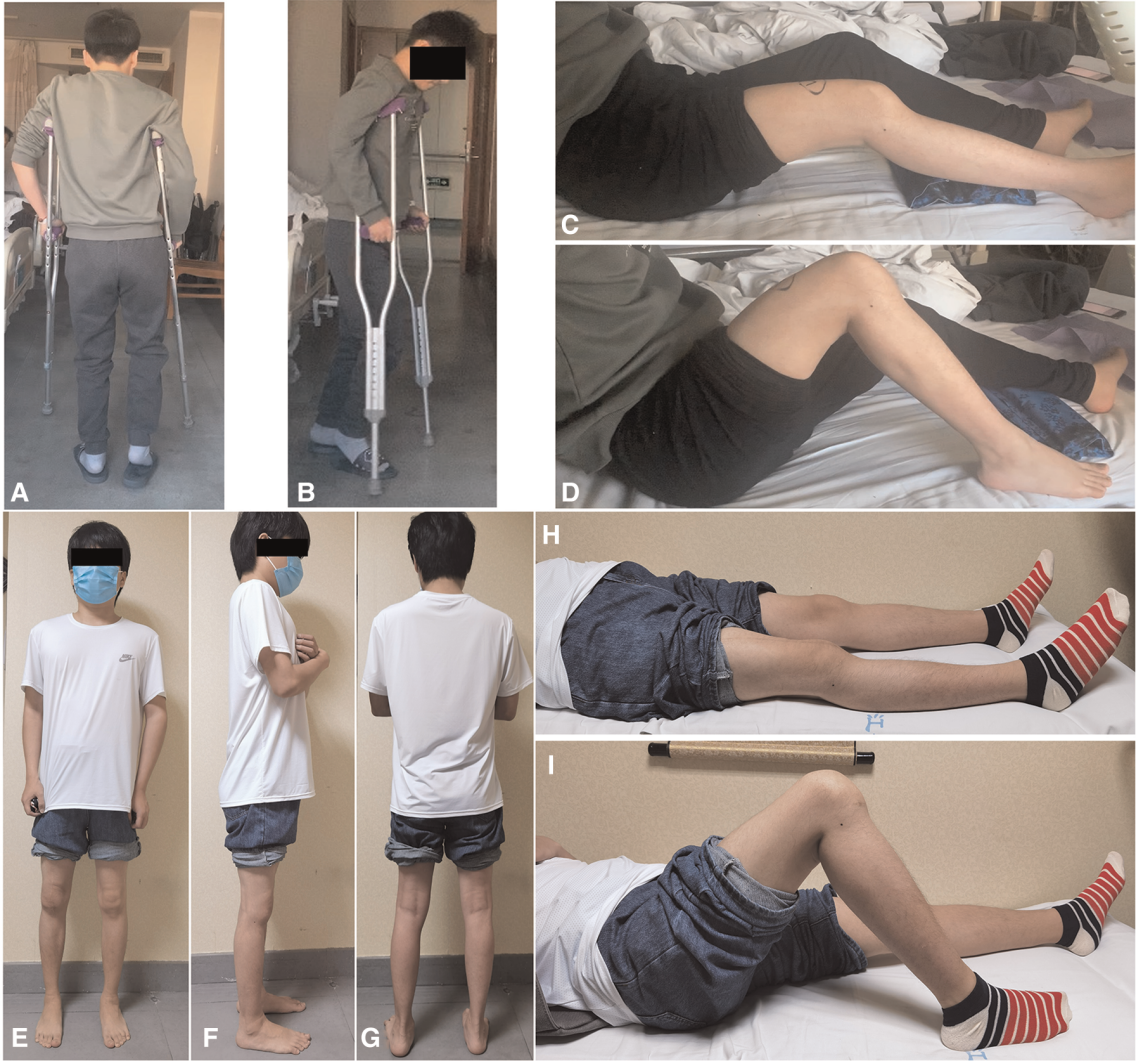
(**A,B**) Preoperative appearance of the right lower limb which shows that the right foot cannot touch the ground; (**C,D**) preoperative range of joint motion (20°–90°); (**E–G**) postoperative appearance of the right lower limb and the right foot touching the ground; (**H,I**) postoperative range of joint motion (0°–95°).

**Figure 2:**
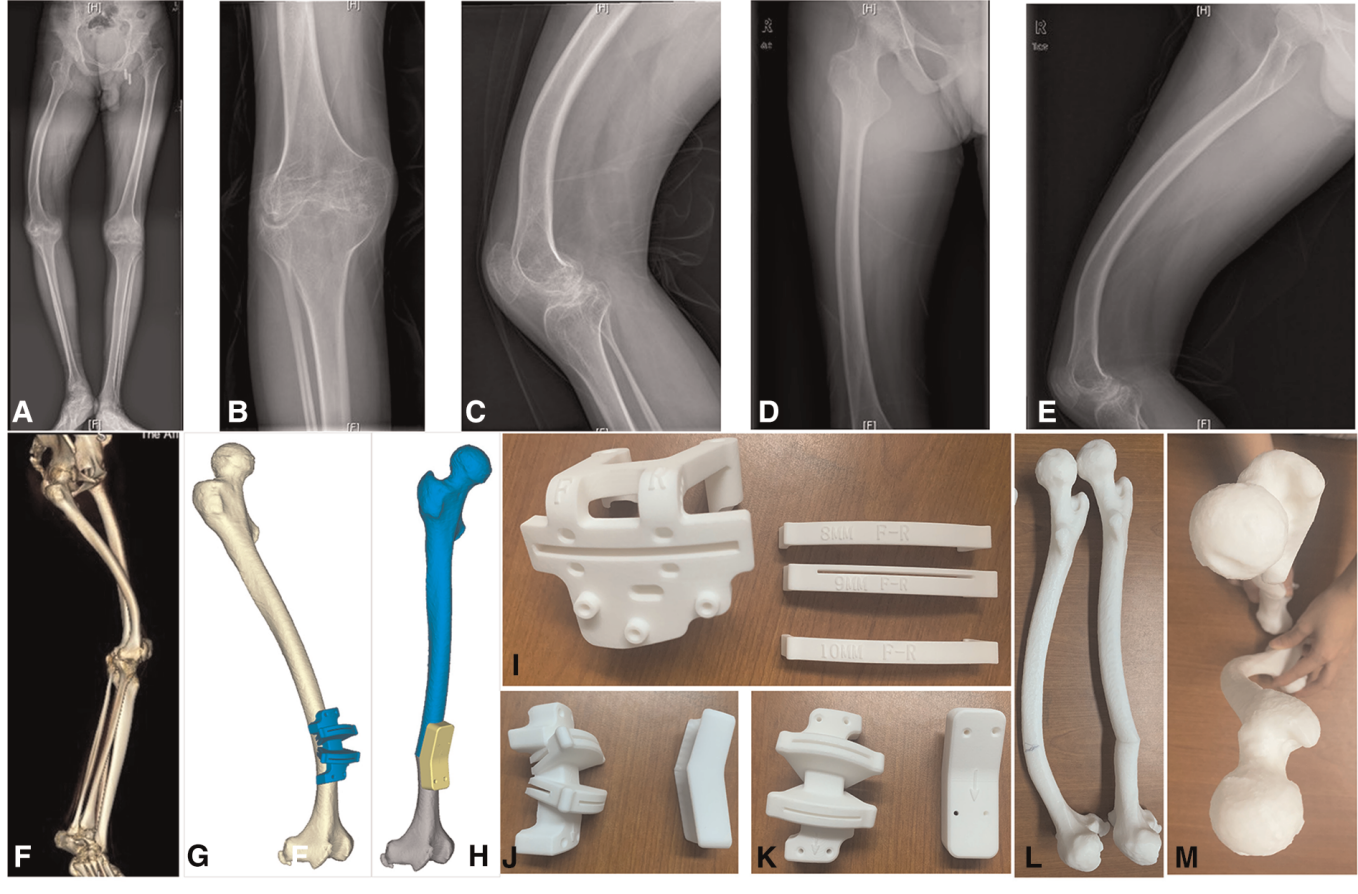
(**A**) Full-length x-rays of both lower limbs (weight-bearing); (**B–E**) anteroposterior and lateral x-rays of the knee joints and femur. The sagittal deformity angle of the femur was 30° (anterior); (**F**) full-length computer tomography film of right lower limb; (**G,H**) computer model of distal femoral osteotomy; (**I**) distal femur osteotomy guide plate in TKA; (**J, K**) osteotomy and reduction guide plate for extra-articular deformities; (**L,M**) models of femur in lateral and overhead positions after the osteotomy.

Preoperative examinations showed the patient had pain in his right knee. His right foot could not touch the ground. The range of joint motion (ROM) was 20°–90°, the visual analogue scale (VAS) was 7 and the Western Ontario and McMaster Universities Osteoarthritis Index (WOMAC) was 106 (Figure 1, Table 2). Other systemic examinations were normal. X-ray and CT showed that the patient's right knee joint space was narrow, with a right femoral flexion deformity (30°, sagittal, anterior) (Figure 2).

**Table 2:** Pre- and post-operative VAS, WOMAC score.

	VAS	WOMAC			
		Pain	Stiffness	Difficulty	Total
Pre-opration	7	26	12	68	106
Post-opration	0	0	3	15	18

## Treatment

Considering the cost, risk and degree of deformity, we decided to carry out a TKA combined with a one-stage EAO under the guidance of PSI to correct the deformity and restore joint function. 3D modelling of the full-length CT of the lower limbs was completed by Arigin 3D STS Design Software (Figure 2). Considering of the possible internal fixation failure and implant failure during TKA, we plan to conduct TKA first, and then perform extra-articular osteotomy. The advantage of this scheme is that the extra-articular osteotomy can adjust the MA again after TKA.

The surgery was performed in the supine position. A tourniquet (40 kPa) was used during the operation. The incision site was at the mid-level knee with a medial patellar approach. According to the bony landmarks of the distal femur, the distal femoral osteotomy was completed by PSI. Neutral tibial resection without PSI was performed for the proximal tibia. External rotation resection (3°) and “4 in 1” guide resection were performed at the 90° position of knee flexion. The patella was not resurfaced, but osteophyte cleaning and denervation were achieved. A cement implant containing vancomycin was placed (PS implant, Stryker Scorpio NRG Knee System).

**Figure 3:**
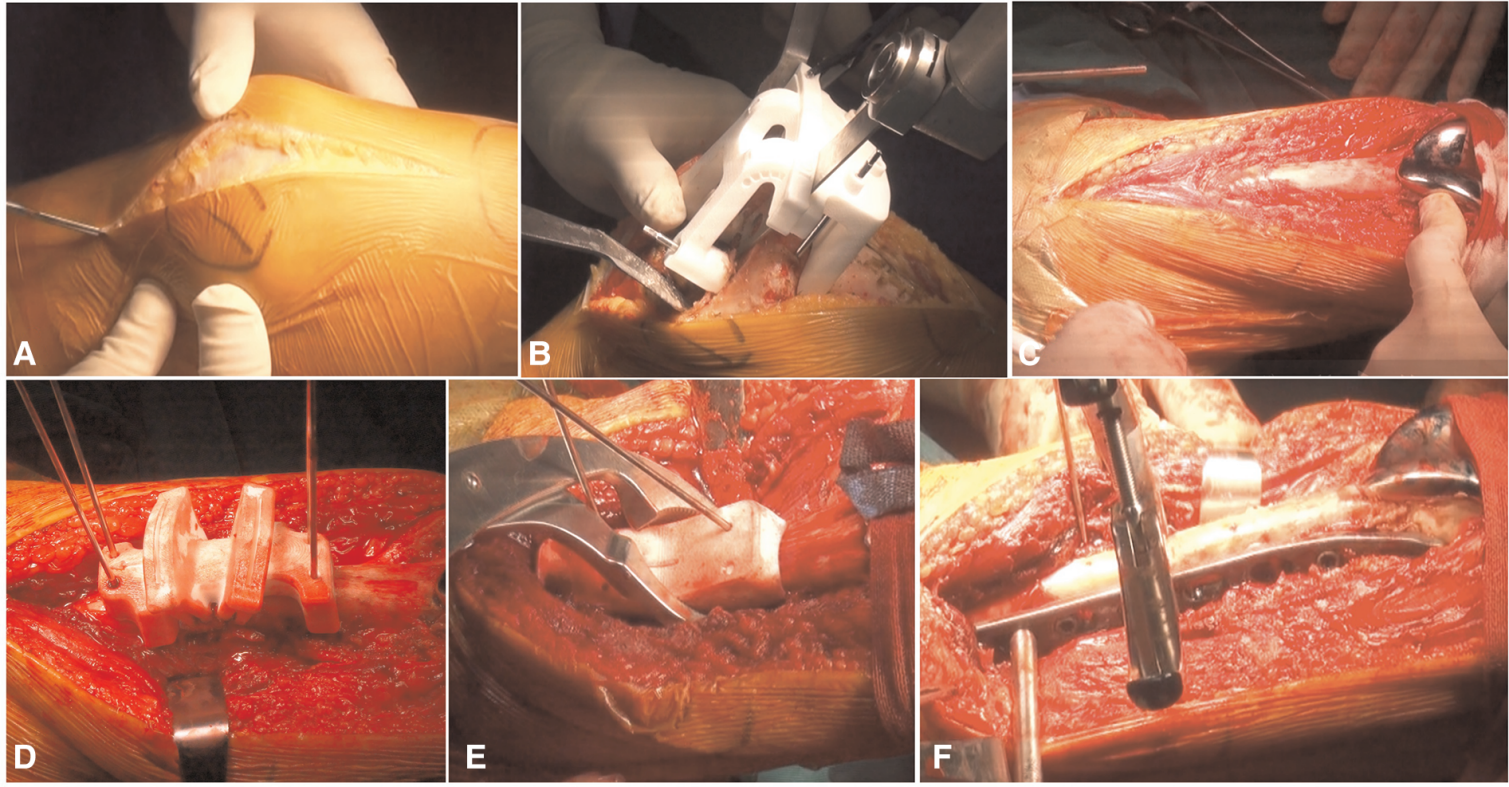
(**A**) Total knee arthroplasty approach; (**B**) the distal femoral osteotomy was guided by PSI; (**C**) the incision was lengthened; (**D**) the femoral shaft osteotomy by patient-specific cutting guide; (**E**) femoral shaft closed reduction was guided by femoral reduction guide plate; (**F**) fixation of osteotomy site.

The incision was extended approximately 15 cm along the proximal femur. Exposure of the deformed segment and bony landmark of the distal femur was made along the lateral femoral muscle space. The PSI was placed in the correct position. A closed osteotomy (30°) was performed on the front of the femur, and the posterior hinge was cut off. A femoral reduction plate was used to fit the osteotomy ends of the femur, which were fixed temporarily by Kirschner wires. After removing the femoral reduction guide plate, a femur shaft locking compression plate (Wego) was placed on the lateral side of the femur. The tourniquet was loosened, and the bleeding was stopped at the visible bleeding points. Layer by layer, the incision was sutured. The joint cavity was injected with 50 ml of normal saline containing 0.8 g of tranexamic acid after the congestion of the joint cavity was removed. The knee joint was bandaged with cotton pads and elastic bandages. After returning to the ward, the knee was immediately iced for 5 days (Figure 3).

We followed our haematological management protocol, which involves 80%–100% correction of Factor VIII before surgery, with the same dose repeated after 8 h. Daily correction of 80%–100% was provided twice a day for 2 days. Then, the patient was evaluated on the third day by the haematologist, and the dose was modified to a 40%–80% correction daily for 5 days. From the 8th day, supplementation with at least 30% correction was performed before each manipulation and rehabilitation training. We performed inhibitor tests before surgery, 3 days after surgery, 1 week after surgery, and before discharge because of the positive history for inhibitors.

## Outcome and follow-up

**Figure 4:**
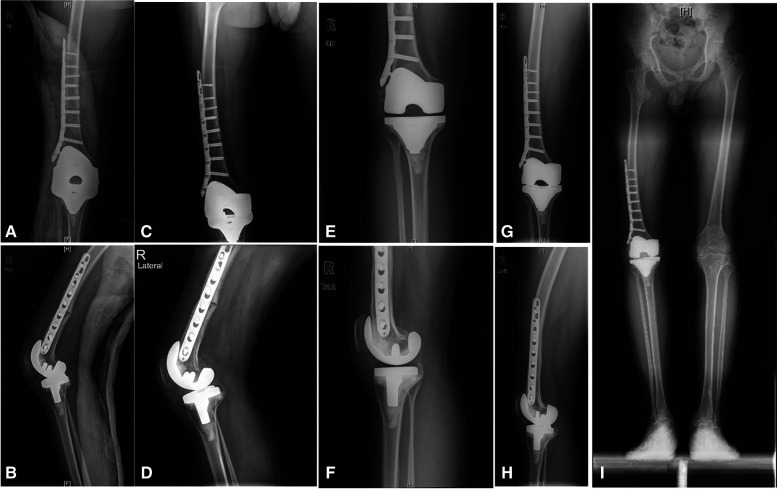
(**A,B**) Postoperative knee and femur x-rays at 3 days; (**C,D**) postoperative x-rays at 7 months; (**E–H**) postoperative x-rays at 23 months; (**I**) full-length x-rays of both lower limbs in weight-bearing positions at 23 months after surgery.

For this patient, we developed an individualized rehabilitation plan. On the first day after the operation, the rehabilitation physician and physical therapist began to intervene, guiding the patient to perform active foot extensions and leg raising exercises under the protection of a brace. From the 3rd day after surgery, active knee flexion and extension exercises and partial weight-bearing were allowed. A continuous passive motion machine was used under the guidance of a rehabilitation specialist. The patient could completely bear weight beginning in postoperative week 12. Within 30 min after the rehabilitation exercise, an ice compress was applied to the surgical site to reduce bleeding and pain. The last follow-up showed that the knee function was good, the VAS was 0, the WOMAC was 18 (Table 2) and the ROM was 0°–95° (28 months) (Figures 1, 4).

## Discussion and review of literatures

We reported a haemophilia patient with inhibitors and end-stage HA who underwent a TKA and osteotomy for severe femoral side deformities with the aid of PSI synchronously.

Haemophilia patients develop end-stage arthritis at a younger age than ordinary arthritis patients. When nonsurgical treatment does not provide satisfactory pain relief and functional improvements, WFH recommends surgical intervention (1). TKA in haemophilic patients is challenging because of the special features of haemophilic patients compared to primary KOA, such as tendencies for bleeding, abnormal bone structures, osteopenia, and a poor soft tissue condition (7).

Patients with FVIII/FIX inhibitors face a higher risk of bleeding during treatment and higher treatment-related costs (8, 9). Despite good results among haemophilia patients with inhibitors (10–12) surgical intervention for haemophilia patients with inhibitors, especially those with high response inhibitors, is limited to emergency treatment of life- or limb-threatening clinical conditions (13). There are different strategies for performing surgical procedures in inhibitor patients (1). The eradication of antibodies through immune tolerance induction (ITI) is still the preferred management strategy for treating inhibitor patients, such as the Bonn scheme, the Van Creveld scheme and the Malmö scheme (14). However, some patients are unresponsive to ITI (15). The inhibitor concentration in this patient was <5.0 BU, which is a low-responding inhibitor. In some patients with low-response inhibitors, the inhibitor would disappear by itself within 6 months (14, 16). It has been proven to be useful for us to control bleeding by eliminating persistent inhibitors by the Van Creveld scheme, which strengthens the perioperative management of coagulation factors and the detection of inhibitors.

EAD was the main surgical challenge for this patient. EAD is mostly secondary to fracture malunion, metabolic bone disease and congenital malformations, accounting for 12% of TKAs (17). The incidence of nonsurgical treatment after fractures in haemophilia patients is higher than that in nonhemophilia patients, which may increase the risk of an EAD. The TKA of knee with EAD can be completed by intra-articular osteotomy, primary or secondary extra-articular osteotomy. Intra-articular osteotomy has the advantages of short operation time, less trauma, quick recovery and fewer complications, but it is limited by the angle of deformity. Although extra-articular osteotomy is not limited by the angle of deformity, there are risks of poor or nonunion of the osteotomy site, increased operation time and cost, delayed rehabilitation and increased infection. For HA patients with severe EAD, simultaneous EAO and TKA can reduce costs and accelerate the return to normal life (6). However, a prolonged operation time and increased tissue damage will increase the risk of bleeding and infection (18, 19). Simultaneous osteotomy and TKA could increase the infection rate, although there are no studies that address this question. We routinely use vancomycin-loaded cement to prevent infection. In addition, we take measures such as supplementation with factor VIII, the use of tourniquets, maintain adequate haemostasis, use tranexamic acid retention perfusion (20), and use compression bandaging and local ice to prevent perioperative blood loss during TKA.

PSI is of great significance for TKA with severe EAD because the osteotomy guide cannot be accurately positioned. Regardless of single-plane or multiplane deformities, PSI can plan the angle and direction of the osteotomy using computer guidance to reduce errors caused by visual observation and operator experience. Lv et al. (6) used PSI to complete 9 cases of TKA with EAD on the femoral side. The follow-up results showed that PSI could effectively restore the lower limb line of force, simplify the operation process, and shorten the operation time.

After orthopaedic surgery, patients should undergo rehabilitation as early as possible, including haemophilia patients. The rehabilitation plan should be jointly decided by a team of haematology specialists, rehabilitation specialists, physical therapists and orthopaedic specialists. Postoperative rehabilitation can help with functional recovery. Inappropriate rehabilitation programs and coagulation factor concentrations will increase the risk of bleeding, which will seriously affect the postoperative joint function and rehabilitation progress (8, 21). In our opinion, the minimum concentration of the coagulation factor should be maintained above 30% during rehabilitation, even if the patient has been discharged from the hospital.

Inhibitor management, surgical planning of HA with severe EAD, individualized rehabilitation and perioperative bleeding risk management were difficulties in the treatment of this haemophilic patient. We consulted haematology experts to complete the cleaning of inhibitors and guide perioperative management of the coagulation factors. With the help of PSI, the TKA and EAO were completed at the same time. PSI can reduce intraoperative bleeding and infection by reducing the difficulty of intraoperative localization and the operation time. Under the guidance of rehabilitation experts and physiotherapists, individualized rehabilitation plans can reduce the risk of bleeding and fracture and obtain good joint function. Since this article is a single case report, the postoperative efficacy and safety of this approach need to be confirmed by a study using a larger sample size.

## Conclusion

Regular haematology management can reduce the perioperative bleeding risk in haemophilic patients treated with inhibitors. With PSI, haemophilic patients who have severe EAD can complete TKA and EAO at the same time, thereby obtaining good postoperative joint function.

## Data Availability

The original contributions presented in the study are included in the article/Supplementary Material, further inquiries can be directed to the corresponding author/s.
